# Temperature Dependence of a Thermosensitive Nanogel: A Dissipative Particle Dynamics Simulation of PNIPAM in Water

**DOI:** 10.3390/ijms27031241

**Published:** 2026-01-26

**Authors:** Daniel Valero, Francesc Mas, Sergio Madurga

**Affiliations:** Department of Material Science and Physical Chemistry, Institute of Theoretical and Computational Chemistry (IQTC), University of Barcelona (UB), 08028 Barcelona, Spain; danivalero@ugr.es

**Keywords:** computer simulations, dissipative particle dynamics, thermosensitive nanogel, PNIPAM, volume phase transition

## Abstract

Thermosensitive nanogels undergo a volume phase transition in response to temperature changes, making them promising candidates for applications, such as water pollutant remediation and drug delivery. In this study, we investigated the thermosensitive volume phase transition of a neutral poly(N-isopropylacrylamide) (PNIPAM) nanogel using coarse-grained dissipative particle dynamics (DPD) simulations conducted using ESPResSo software with varying bead volumes. Langevin dynamics simulations were employed to compare the results. In DPD simulations, water is explicitly treated, whereas in Langevin dynamics, it is treated implicitly, and hydrophobic interactions are represented by an attractive potential between monomer beads. Our results, including the radius of gyration and various radial distribution functions, revealed a clear volume phase transition as the temperature varied, transitioning from an expanded state to a collapsed state. Notably, the volume phase transition observed in Langevin simulations is attributed to the attractive potential between the PNIPAM monomers, whereas in the DPD simulations, it arises from implicit hydrophobic interactions, obviating the need for an additional attractive potential between the monomer beads. This implicit hydrophobic effect originates from the temperature dependence of the Flory–Huggins interaction parameter.

## 1. Introduction

Nanogels, composed of crosslinked polymeric chains forming a network, exhibit dimensions on the sub-micron scale and demonstrate a responsive behavior to various stimuli, including changes in pH, ionic strength, and temperature [1,2]. Among these, thermosensitive nanogels display a remarkable volume phase transition in response to temperature variation.

This volume phase transition involves a shift from a swollen to a collapsed state within the nanogel structure, primarily driven by temperature-dependent hydrophobic interactions mediated by the surrounding solvent. The mechanism underlying these interactions can be attributed to the mixing entropy of the constituent monomers of the nanogel in the solvent medium. Depending on the nature of the interactions between the nanogel monomers and solvent, two distinct types of volume phase transitions can be observed.

The first scenario involves the transition from a collapsed state at lower temperatures to a swollen state at higher temperatures, characterizing nanogels that exhibit an upper critical solution temperature (UCST) [3]. In contrast, in the second scenario, the nanogels shifted from a swollen state at lower temperatures to a collapsed state at higher temperatures, indicating a lower critical solution temperature (LCST) [3,4,5,6,7]. This behavior makes thermosensitive nanogels promising candidates for a multitude of applications, ranging from sensors [8] to water purification [9,10] and drug delivery systems [11,12,13,14]. Nonetheless, to fully exploit their potential in these domains, it is imperative to meticulously calibrate their swelling and deswelling characteristics to satisfy precise specifications. Consequently, understanding the influence of various stimuli on the volume phase transitions of thermosensitive nanogels is of paramount importance for customizing their properties to align them with diverse application requirements.

Computational studies employing both full-atom [15,16,17,18,19,20,21,22,23,24,25] and coarse-grained [26,27,28,29,30,31,32,33,34,35,36,37,38,39,40,41,42,43,44,45] thermosensitive polymer models have been conducted in this context.

Full-atom models have been used to study the LCST transitions of linear-chain thermosensitive polymers and copolymers. These studies [15,16,17,18,19,20,21,22,23,24,25] explored various aspects, including the effect of the linear chain length on the LCST transition [15], the effect of stereochemistry on the thermosensitive response [17], and the influence of different water models on the critical temperature of the volume phase transition [23,25].

Coarse-grained models have been used to study thermosensitive polymers under various environmental conditions, including pH [33,35,40,45], ionic strength [35,40], and chain length [29,30,32]. Coarse-grained approaches with an implicit solvent, such as Langevin dynamics [29,30], Brownian dynamics [33], and Monte Carlo simulations [26,32,35,38,40,45], have been employed to examine the volume phase transition in thermosensitive gels. In these simulations, temperature-dependent hydrophobic interactions with the solvent were typically represented by the attractive potential between the monomer beads. While some potentials are only applicable to specific temperatures for which they are parameterized [26,38,45], others are temperature-dependent [27,28,32,35] and can be used across a range of temperatures in the simulations.

In this study, we employed dissipative particle dynamics (DPD) [39,44,46,47,48,49,50] by integrating specific volume parameters for beads to represent nanogel monomers and solvent molecules to simulate the swelling behavior of the nanogels. DPD simulations are coarse-grained techniques in which the solvent is represented by beads encapsulating multiple solvent molecules. The interactions among these beads included conservative, dissipative, and fluctuating forces, and the beads were characterized as soft spheres. Consequently, DPD simulations explicitly consider solvent particles and their interactions, enabling longer time-steps than all-atom molecular dynamics simulations. The explicit treatment of the solvent in the DPD simulations ensures the accurate simulation of system hydrodynamics [46,47], thereby facilitating the manifestation of hydrophobic interactions between the solvent and nanogel monomers. This makes DPD a suitable method for investigating the volume phase transition of thermosensitive nanogels because such transitions originate from hydrophobic interactions.

Different systems have been investigated using DPD simulations, including micelle formation [51], vesicle formation [52], microgels adsorbed at liquid–liquid interfaces [53], pH-dependent copolymer self-assembly [54], brush arrays [55], biological membranes [56], membrane structure [57,58], structure, and stress distribution of amphiphilic bilayers [59], thermosensitive linear polymers [31], thermosensitive brush arrays [34], thermosensitive polymeric micelles [36,42], thermosensitive copolymers, [41,43] and thermosensitive microgels [21,37,39,44]. Previous DPD studies of thermosensitive polymers typically employed monomers and water beads of a uniform volume. In the case of thermosensitive microgels, the volume phase transition is often studied using the radius of gyration [39] or porosity [37] as a function of the DPD repulsion parameter between the water and monomer beads. In this study, we present the radius of gyration of the nanogel as a function of temperature, which facilitates a straightforward comparison with the experimental data.

There are different DPD parameterization methods that use beads of different volumes, including those developed by Kacar et al. [60] and those used to develop a DPD force field with beads of varying volumes [61]. Using beads of different volumes allows for more accurate consideration of the steric effects between the beads. For thermosensitive nanogels, steric effects become increasingly important as the nanogel collapses. Therefore, it is advantageous to use beads of varying volumes to capture the collapsed state more accurately.

The parameterization method employed to simulate the DPD beads of different volumes was that of Kacar et al. [60]. In this DPD parameterization, the Flory–Huggins interaction parameter [62] plays a predominant role in the temperature-dependent hydrophobic effect. Therefore, it is necessary to determine and tune these parameters carefully to obtain accurate quantitative results. This parameter tuning is not only important in the case of the Flory–Huggins parameter, but also for all the force field parameters. In the DPD case, the Flory–Huggins parameter is the most important because it determines the transition temperature. In this work, the Flory–Huggins parameter was approximated by fitting the experimental data of microgels to the well-known expression of the temperature dependence of the Flory–Huggins interaction parameter [63,64]. To the best of our knowledge, this study is the first to investigate the volume phase transition of thermosensitive nanogels with DPD simulations of varying bead volumes and by approximating the Flory–Huggins parameter using a fit to the Flory–Huggins equation.

Therefore, the aim of this study was to parameterize DPD interactions to model and simulate a neutral poly(N-isopropylacrylamide) (PNIPAM) thermosensitive nanogel at different temperatures, with specific volumes assigned to the monomer and water beads. This study also aimed to introduce DPD simulations with beads of different volumes as a technique to determine the volume phase transition of thermosensitive nanogels. For the simulation of nanogels with acid–base functional groups, the nanogel is not neutral, and electrostatic interactions must be implemented. In such cases, various methods exist for incorporating charges into the DPD models, including implementing charges as Slater-type charges [65] and using Gaussian charge densities [66].

PNIPAM undergoes a volume phase transition characterized by an LCST (typically in the range of 300–305 K [3]), making it a versatile polymer for numerous applications. These applications include smart actuators, colloidal crystals, and smart hydrogels, which are used in drug delivery systems. Given that the volume phase transition temperature of PNIPAM aligns with the human physiological temperature, it serves as an ideal thermosensitive gel for drug delivery [67].

The LCST transition of PNIPAM has been extensively studied using experimental investigations [22,68,69,70,71,72] and full-atom simulations [15,16,19,20,22,23,24,25] of the PNIPAM linear chains. At temperatures below the LCST, the PNIPAM hydration layer formed a cage-like structure, with water molecules surrounding the PNIPAM monomers forming hydrogen bonds with each other and the amide groups of the monomers [16]. When the temperature rose above the LCST, the hydrogen bonds in this cage-like structure, which stabilize the expanded state of PNIPAM, began to break [16,17,20,22,23,24,70]. This increased the entropy of water, which triggered the collapse of the PNIPAM polymer. During this collapse, hydrogen bonds formed between the amide groups of different monomers [16,17,22,23,24,71], further stabilizing the collapsed structure.

The critical volume phase transition temperature of PNIPAM linear chains varies depending on the number of monomers per chain [15,69], tacticity of the linear chain [17,68], and concentration of linear chains [69].

To investigate the volume phase transition of PNIPAM, DPD and Langevin dynamics simulations were conducted using ESPResSo 4.2 software [73,74]. In Langevin dynamics simulations, the force field was adjusted to investigate the effect of charged beads on the microgel. This force field includes a temperature-dependent attractive potential between uncharged monomer beads to model the hydrophobic effect. A force field was developed by Quesada-Pérez et al. [33].

Langevin dynamics simulations were used to compare the PNIPAM volume phase transition results obtained using a previously developed methodology with those of the methodology proposed for DPD. From these simulations, the radius of gyration of the nanogel and different radial distribution functions were obtained to compare the results obtained using the two methods.

Given the potential interest in studying higher-order polymer structures, the periodic structure of a system can be analyzed using radial distribution functions. The structure factor, which can be obtained through the Fourier transform of the radial distribution functions [75], is useful for examining these periodic structures. However, because our system consisted of an isolated nanogel, higher-order structures were not addressed in this study.

## 2. Results and Discussion

**Figure 1:**
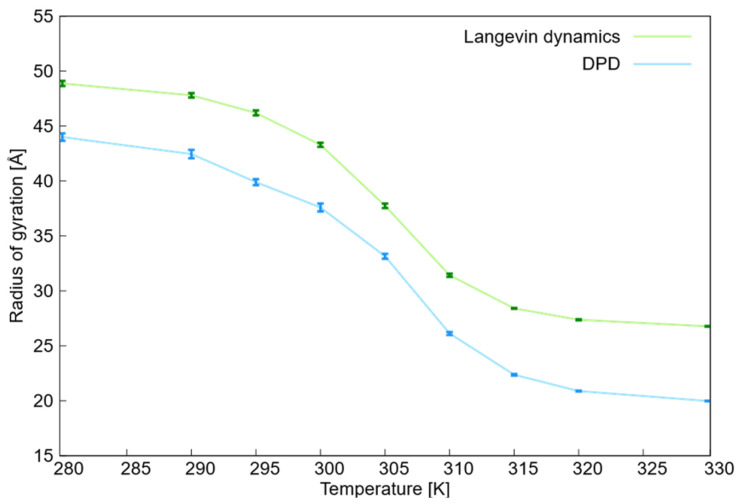
Radius of gyration (Rg) of the PNIPAM nanogel as a function of temperature, comparing DPD (blue) and Langevin dynamics (green) simulations. The plot illustrates the volume phase transition from a swollen state at low temperatures to a collapsed state at higher temperatures, with a critical transition range between 305 and 310 K. Error bars represent the standard error of the mean (SEM) calculated using the method of binning correlated data to ensure statistical reliability. Solid lines connect the mean values and serve as a visual guide.

The investigation of the volume phase transition in the simulated PNIPAM nanogel involved analyzing the radius of gyration (Rg) and radial distribution function. The radius of gyration offers insight into the nanogel size, indicating whether the nanogel is in a condensed or swollen state. Meanwhile, the radial distribution function between the nanogel’s center of mass and the water beads assists in determining the swelling state and presence of water beads within the nanogel structure. To investigate the volume phase transition of the simulated PNIPAM nanogel, both the radius of gyration and radial distribution function were analyzed. The estimated radius of gyration of the PNIPAM nanogel in the temperature range of 280–330 K, obtained from DPD and Langevin dynamics simulations, is illustrated in Figure 1, with uncertainties derived from binned correlated data. To ensure statistical reliability and address the scattered nature of DPD results, uncertainties were determined by binning correlated data, grouping simulation points into independent blocks to obtain a robust standard deviation and a robust estimation of the standard error of the mean (SEM). As shown in Table 1, the error bars for the radius of gyration decrease significantly in the collapsed state, confirming the stability of the nanogel structure at high temperatures. Thus, increased temperatures lead to more compacted structures with reduced structural fluctuations.

The DPD and Langevin simulations showed a similar evolution of the radius of gyration as a function of temperature. However, a discrepancy was observed in the radius of gyration, with the Langevin dynamics simulations yielding larger values than those of the DPD simulations.

The smaller radii of gyration in the DPD simulations can be attributed to the soft-sphere nature of the beads. Unlike the hard-sphere Weeks-Chandler-Andersen (WCA) potential in Langevin dynamics that prevents particle interpenetration, the DPD potential allows for partial overlap. This flexibility enables a more compact densification of the polymer network during collapse, which is reflected in the shift in the *g*(*r*) peaks toward shorter distances as temperature increases.

This indicates that the differences observed in the results stem from the fact that the parameters of the two force field models do not directly correspond to each other. In DPD simulations, the primary parameters are the maximum repulsive forces between pairs of particles (${Fmax}_{ij}$), whereas in Langevin dynamics, the corresponding parameters include the effective radius of the monomer, depth of the potential, and various parameters of the hydrophobic attractive potential. For more information on the parameters of the Langevin dynamics of non-bonded potentials, please refer to Supplementary Section S2.1.

Uncertainties in the radius of gyration data (Figure 1 and Table 1) were determined by binning the correlated data. This involves grouping the data into blocks of increasing size and calculating the standard deviation for each block size. These standard deviations were plotted against the number of points per block, revealing a flat region. The final uncertainty was obtained by averaging the standard deviations within this flat region, which typically includes data blocks of 12–25 independent points in the simulations. The DPD simulation results related to water beads are provided in Supplementary Section S4.4.

**Figure 2:**
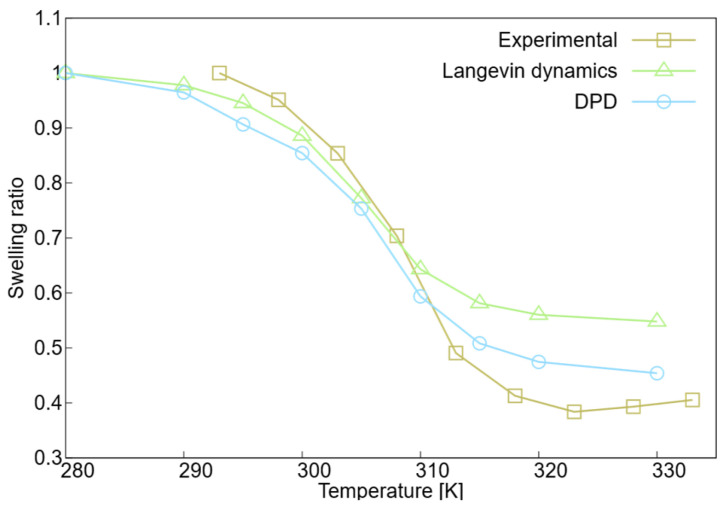
Swelling ratio versus temperature for experimental data (squares) of a microgel with 8 monomers per chain and 0 acrylic acids [33], Langevin dynamics (triangles), and DPD simulations (circles) of the PNIPAM nanogel. For all data sets, the lowest temperature was used as the reference temperature.

The swelling ratio was employed as the primary metric for comparison to account for the inherent scale disparity between experimental samples and computational models. While experimental benchmarks are derived from PNIPAM microgels, which exist on a larger scale, our simulations focus on an isolated nanogel consisting of 439 monomer beads. Because a direct comparison of absolute dimensions is not feasible across these different length scales, the swelling ratio, calculated as the size at a given temperature relative to the size at the lowest reference temperature, provides a normalized measure of the proportion of size change. The swelling ratio shown in Figure 2 for the experimental data was calculated as the ratio between the size of the hydrodynamic radius at any temperature and that at the reference temperature, which was chosen as the lowest temperature in each dataset [76]. For the simulations, as an approximation, the swelling ratio was determined using the radius of gyration as a measure of nanogel size, $Rg(T)/Rg(T_{ref})$. For the experimental data [33], the diameter of the microgel was used as the size measure ($d(T)/d(T_{ref})$). The swelling ratio of the experimental microgel was smaller than that of the simulations, indicating that the microgel collapsed more at high temperatures than the simulated nanogel did. When comparing the two simulation techniques, the DPD simulations exhibited a smaller swelling ratio at higher temperatures than Langevin dynamics. These observations indicate that the DPD simulations yield results closer to the experimental data, particularly at high temperatures when the nanogel collapses. Thus, the explicit treatment of water in DPD allows for a more accurate reproduction of the swelling ratio in the collapsed state compared to implicit Langevin models. This suggests that the explicit exclusion of solvent beads from the nanogel core is essential to capture the high-density packing observed experimentally at temperatures exceeding 315 K.

Because the collapse of PNIPAM polymers involves the breaking of hydrogen bonds between water molecules and those between water molecules and the amide groups of PNIPAM, and the formation of hydrogen bonds between the amide groups of different monomers, it is challenging to obtain detailed information about the collapse mechanism using DPD simulations that do not account for hydrogen bonds. To address this, Vishnyakov et al. [77] proposed the use of Morse potential to model hydrogen bonding in DPD simulations. Adding this hydrogen bonding model would help determine results that are closer to the experimental results. Nevertheless, before adding hydrogen bonding to DPD, the possibility of better determining the DPD parameters should be investigated.

**Figure 3:**
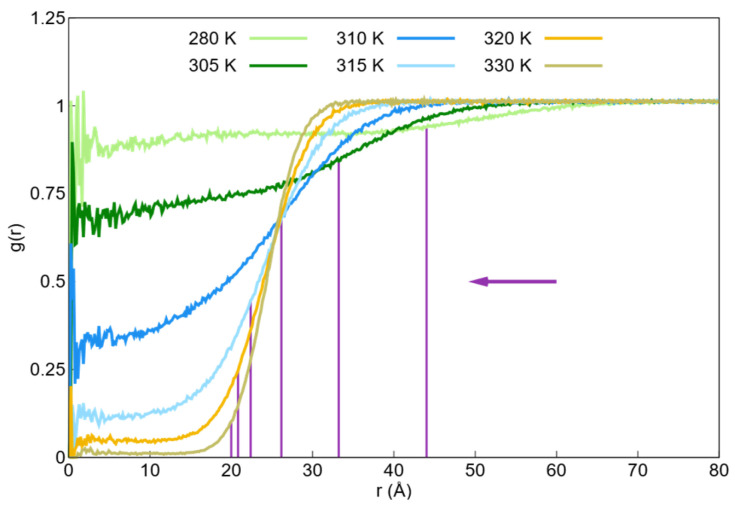
Radial distribution functions (g(r)) between the center of mass of the PNIPAM nanogel and water beads in the temperature range of 280–330 K. The vertical purple lines indicate the mean radius of gyration (Rg) for each temperature, illustrating the structural contraction of the network. The horizontal purple arrow points in the direction of increasing temperature, highlighting how the nanogel’s core becomes progressively hydrophobic and excludes water beads as it transitions from a swollen to a collapsed state.

The radial distribution functions between the center of mass of the studied nanogel and the water beads in the temperature range of 280–330 K are shown in Figure 3. This radial distribution function cannot be measured experimentally; however, it is useful for identifying low-density regions. At 280 K, the number of water beads inside the nanogel decreased as the temperature increased. This observation illustrates the temperature-dependent hydrophobic interaction between water and monomer beads as well as the transition from a swollen state to a collapsed state.

It can be observed that the PNIPAM nanogel undergoes a volume phase transition from a swollen state at temperatures below the critical temperature to a collapsed state at temperatures above the critical temperature. The critical temperature range is 305–310 K. This finding aligns with the experimental values reported for the critical temperature of PNIPAM microgels, which was approximately 310 K [33,64].

When comparing the critical temperature of the volume phase transition for the simulated nanogel with that of the experimental linear chains containing eight monomers at various concentrations, we observed that the critical temperature for the nanogel was lower than that for the linear chains at different concentrations. Specifically, Shan et al. [65] reported that the critical temperature decreases from approximately 343 K at a concentration of approximately 3 mg/mL to approximately 317 K at a concentration of 10 mg/mL. They also noted that, for linear chains with 28 monomers per chain, the critical temperature tended to plateau as the concentration increased. This suggests that the PNIPAM nanogel exhibits a critical temperature like that of a solution with a high concentration of linear chains, provided that both systems have the same number of monomers per chain.

**Figure 4:**
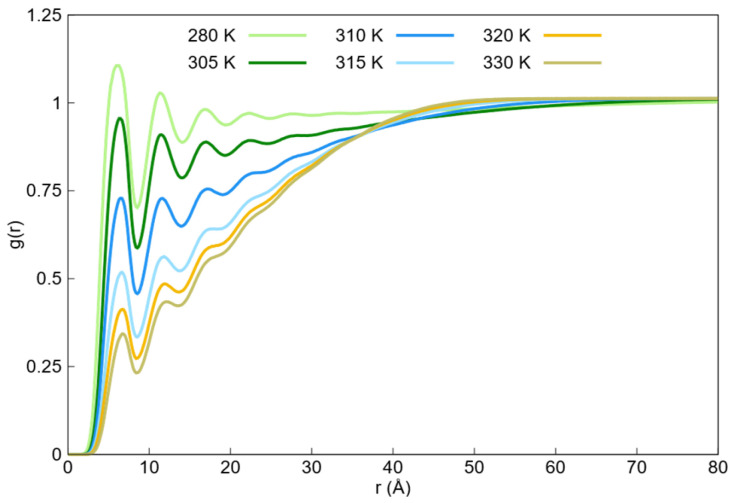
Radial distribution functions (g(r)) between monomer beads and water beads obtained from DPD simulations in the temperature range of 280–330 K. The plots reveal a temperature-dependent trend in the first solvation shell. As the temperature rises, the intensity of the first peak significantly decreases, indicating a reduction in the population of water beads surrounding the PNIPAM monomers.

The radial distribution functions of the DPD simulations between the water and monomer beads within the temperature range of 280–330 K are shown in Figure 4. Additional radial distribution functions of water beads with themselves obtained from DPD simulations are shown in Supplementary Figure S1. It can be observed from Figure 4 that the first solvation shell exhibits a temperature-dependent trend, with a decreasing population as the temperature increases. This decline in the first peak can be attributed to the transition from the swollen state to the collapsed state, because monomer beads are expected to be less solvated by water beads in the collapsed state. Thus, the reduction in the intensity of the first solvation shell (Figure 4) provides a direct visual representation of the thermodynamic exclusion of the solvent. This process is driven by the temperature-dependent Flory–Huggins interaction parameter, which increases the repulsion between water and monomer beads (Fmax*_wm_*), making the hydration layer energetically unfavorable at temperatures above the LCST.

**Figure 5:**
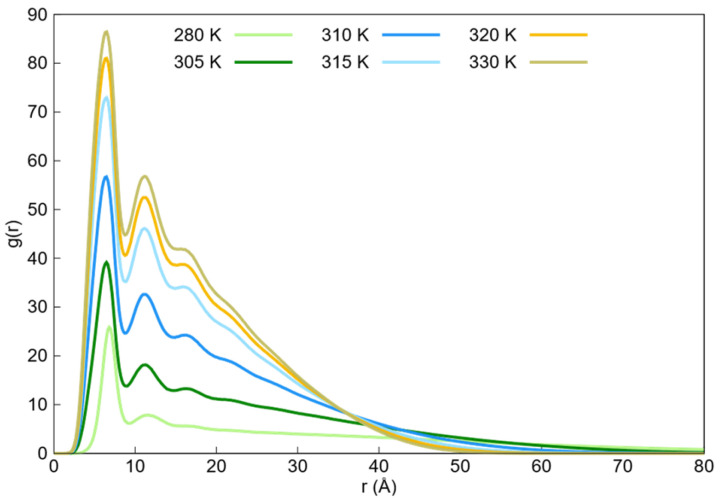
Monomer–monomer radial distribution functions (g(r)) obtained from DPD simulations across the 280–330 K temperature range. The plot illustrates the structural densification of the PNIPAM network during the volume phase transition. As the temperature increases, the intensity of the peaks rises significantly, reflecting the transition from a swollen state to a highly aggregated collapsed state.

**Figure 6:**
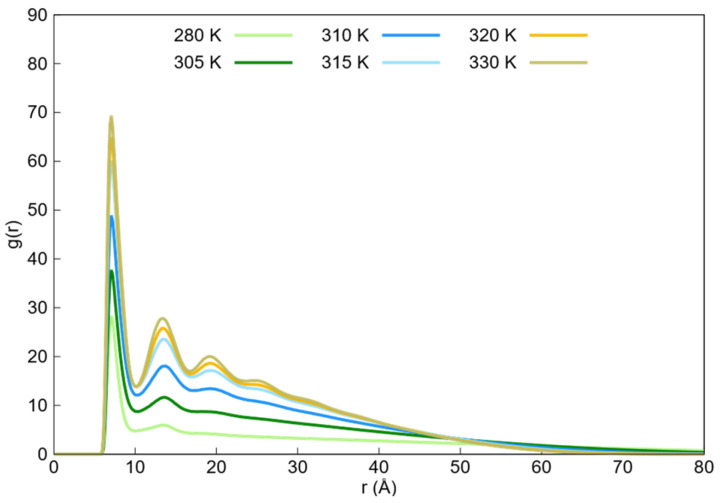
Monomer–monomer radial distribution functions (g(r)) obtained from Langevin dynamics simulations across the 280–330 K temperature range. The profiles illustrate the structural densification of the PNIPAM nanogel driven by a temperature-dependent hydrophobic attractive potential.

The radial distribution functions of the monomer beads obtained from the DPD simulations within the temperature range of 280–330 K are shown in Figure 5. The corresponding radial distribution functions from Langevin dynamics for monomer beads within the same temperature range are shown in Figure 6.

In both figures, the radial distribution functions tend toward zero at long distances because of the absence of monomers, with the rate at which they approach zero increasing with the higher aggregation state of the nanogel. The tails of these profiles were used to approximate the maximum nanogel diameters at each temperature.

The observed trend reflects the transition from the swollen state to the collapsed state as the temperature increases. Furthermore, for the DPD radial distribution functions, two pronounced peaks were observed at the beginning of these functions. The center of the first peak shifted from 6.8 to 6.5 Å, and the second peak shifted from 11.3 to 11 Å as the temperature increased. In contrast, the Langevin dynamic radial distribution functions also showed two prominent peaks around 7 and 13.4 Å, but these peaks did not shift with increasing temperature. Additionally, a third peak appeared around 19.3 Å as the temperature increased. In this context, the first and second peaks corresponded to the first and second neighbors, respectively, while the third peak in Langevin dynamics corresponded to the third neighbor.

It is worth noting that the value of these peaks was high because the monomers were bound by bonds in a localized region of space, resulting in a high density, as neighboring beads could not be easily separated.

**Figure 7:**
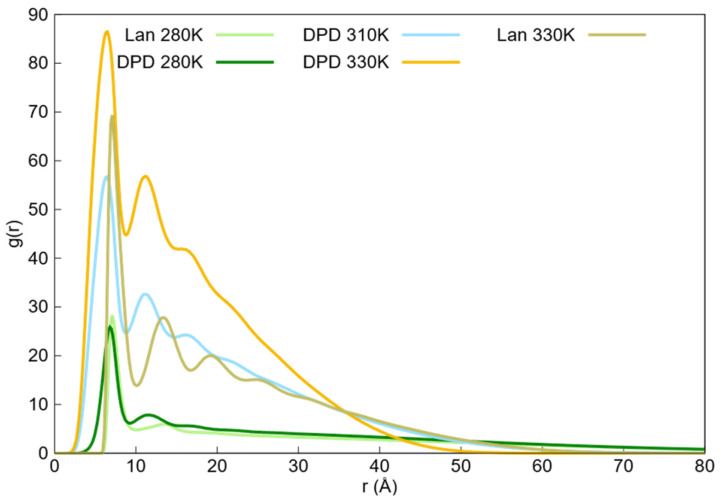
Radial distribution functions of the monomer beads interacting with themselves of Langevin dynamics simulations at 280 K and 330 K, and for DPD simulations at 280 K, 310 K, and 330 K.

To further compare the radial distribution functions of the monomer beads interacting with themselves obtained from the two simulation methods, we plotted these functions at the lowest and highest temperatures for both the simulation techniques, as shown in Figure 7. Additionally, we included the radial distribution function at 310 K from the DPD simulations because the radius of gyration of the Langevin dynamics simulations at 330 K was similar to that of the DPD simulations at 310 K.

Comparing the radial distribution functions of DPD and Langevin dynamics simulations at 310 and 330 K, respectively, it is observed that both tails are almost identical. This similarity was due to the comparable radii of gyration in both simulations.

The first peaks of the DPD simulations were broad and shifted to smaller distances as the temperature increased, whereas the peaks from the Langevin dynamics simulations were sharp, did not shift, and appeared at greater distances than the first DPD peak. For the second peaks, both simulation techniques showed similar widths, but the Langevin dynamics peaks did not shift and occurred at larger distances compared with the second DPD peaks.

The differences in the width of the first peak and the shift in the peaks with increasing temperature arose from the nature of the particles used in the simulations: soft particles in the DPD simulations and hard spheres in the Langevin dynamics simulations. The soft particles in the DPD simulations allowed for some degree of overlap, which could bring the neighboring particles closer together and widen the first peak. As the temperature increased, the increased overlap caused the peak to shift to smaller distances. In contrast, the hard-sphere model used in Langevin dynamics did not allow for overlap, resulting in sharper peaks that did not shift with the temperature. The distinct third peak observed in the Langevin dynamics simulations as the temperature rose and the nanogel collapsed was due to the formation of a clear third neighboring shell. Although a similar third layer of neighbors appeared in the DPD simulations, the peak was less pronounced owing to the overlap of soft particles.

## 3. Materials and Methods

### 3.1. DPD Theoretical Background

In DPD, the total non-bonded $f_{i}$ force acting on each pair of interacting coarse-grained particles consisted of three components: conservative forces $F_{ij}^{C}$, dissipative forces $F_{ij}^{D}$ and random forces $F_{ij}^{R}$.

(1) $$f_{i}=\sum_{j\neq i}^{N}\left(F_{ij}^{C}+F_{ij}^{D}+F_{ij}^{R}\right)$$

The stochastic and dissipative forces in the DPD used in this study were formulated according to Español and Warren [47]. In this formulation, pairwise dissipative and stochastic forces are expressed as follows:

(2) $$F_{ij}^{D}=-\gamma \;\omega^{D}\left(r_{ij}\right)\left({\hat{r}}_{ij}\cdot v_{ij}\right){\hat{r}}_{ij}$$

(3) $$F_{ij}^{R}=\sigma \omega^{R}\left(r_{ij}\right)\theta_{ij}{\hat{r}}_{ij}$$

where $r_{ij}$ is the distance between particles i and j; ${\hat{r}}_{ij}$ is the unit vector between the centers of particles *i* and *j*; $v_{ij}$ is the relative vector velocity between the pair of particles *i* and *j*; γ is the friction coefficient; $\theta_{ij}$ is a white noise variable drawn from a uniform distribution; and σ is the noise strength. γ and σ can be defined for each bead-type interaction, but they are assumed to be the same, regardless of the type of bead interaction. This assumption was made because transport phenomena, such as nanogel diffusion, were not the focus of this study. To obtain more accurate results for the transport phenomena, it is necessary to establish a different friction coefficient for each interaction type. $\omega^{D}\left(r_{ij}\right)$ and $\omega^{R}\left(r_{ij}\right)$ are weight functions that depend on the distance between the particles, and their values are zero for distances greater than the cutoff distance $r_{c}$. A weight function was first proposed by Soddemann et al. [49].

(4) $$\omega^{D}\left(r_{ij}\right)=\left\{\begin{array}{cc} 1, & r_{ij}<r_{c}\\ 0, & r_{ij}\geq r_{c} \end{array}\right.$$

(5) $${\left[\omega^{R}\left(r_{ij}\right)\right]}^{2}=\omega^{D}\left(r_{ij}\right)$$

For the fluctuation–dissipation theorem to hold, the following expression must be satisfied:

(6) $$\sigma^{2}=2\gamma k_{B}T$$

where the value of γ (Equation (6)) used in this study was 4.5 in reduced units $\left(\tilde{\gamma }\right)$, as given by Groot and Warren [48].

The conservative forces arise from a soft, repulsive, and conservative interaction potential,

(7) $$U\left(r_{ij}\right)\;=\left\{\begin{array}{cc} Fmax_{ij}\left(r_{ij}-r_{c}\right)\left(\frac{r_{ij}+r_{c}}{2r_{c}}-1\right) & ;\;r_{ij}\leq r_{c}\\ \;\;\;0 & ;\;\;\;r_{ij}>r_{c} \end{array}\right.$$

where ${Fmax}_{ij}$ is the force parameter for the maximum repulsion strength of the interaction potential. This potential is the standard DPD potential implemented by the ESPResSo software [73,74].

### 3.2. Volume Bead Dependence Parametrization

To model multicomponent systems in DPD, Groot and Warren used the Flory–Huggins solution theory [62]. This theory establishes a relationship between the intercomponent interaction parameter and the Flory–Huggins interaction parameter $\chi_{ij}$. This relationship is derived under the assumption that the intra-component interactions of different beads are equivalent. Consequently, beads representing distinct chemical groups were assigned to the same volume.

In this study, we adopted the parameterization proposed by Kacar et al. [60], which enables the modeling of beads with different volumes:

(8) $$Fmax_{ii}=\frac{p-\rho_{i,pure}k_{B}T}{\alpha \rho_{i,pure}^{2}r_{c}^{4}},\alpha =0.101$$

(9) $$Fmax_{ij}=\hat{F}max_{ij}+\frac{p}{0.0454\left(Fmax_{ii}\rho_{i,pure}+Fmax_{jj}\rho_{j,pure}\right)}$$

(10) $$\hat{F}max_{ij}=\sqrt{Fmax_{ii}Fmax_{jj}}$$

where $\rho_{i,pure}$ is the pure number density of the components, which is $\rho_{w,pure}$ for water and $\rho_{m,pure}$ for the PNIPAM monomers. ${\hat{F}max}_{ij}$ is the neutral interaction parameter, p is the applied pressure that is not related to the physical pressure of the system, α is the scaling factor of the relationship between the excess pressure, ρ is the density number of the system, and $\chi_{ij}$ represents the Flory–Huggins interaction parameter between components. In this study, the $\chi_{ij}$ parameter was obtained for each simulated temperature by fitting the experimental data to the original equation from the dependence of the Flory–Huggins parameter [63,64]. However, it is possible to derive $\chi_{ij}$ from quantum calculations, such as ab initio calculations using the fragment molecular orbital method [78,79,80].

The fitting parameters used to compute the Flory–Huggins interaction parameter at each temperature between the water and monomer beads are given by Equation (11) [62,64].

(11) $$\chi_{ij}\left(T\right)=0.5+A\left(1-\left(\frac{\Theta }{T}\right)\right);A=35.2\Theta =308.3$$

The pressure p was determined by matching the repulsive force parameter ${Fmax}_{ii}$ of water (${Fmax}_{ww}$) in Equation (8), using the parameter obtained from the original DPD parameterization of Groot and Warren [48] (Equations (12) and (13)),

(12) $$Fmax_{ww}=N_{m}\frac{\left(\overset{\sim \;\;\;\;}{k^{-1}}-1\right)}{2\alpha \rho }\frac{k_{B}T}{r_{c}^{4}}$$

(13) $$\overset{\sim \;\;\;\;}{k^{-1}}=\frac{1}{\rho_{w,pure}k_{B}T\kappa_{T}}$$

where $N_{m}$ is the number of water molecules in the water bead, $\overset{\sim \;\;\;\;}{k^{-1}}$ is the inverse dimensionless isothermal compressibility, and $\kappa_{T}$ is the isothermal compressibility of the water [78]. Given that the simulated mixture mostly comprised solvent beads with only a few monomers, its compressibility closely approximated that of water. This ensures that the pressure matches the isothermal compressibility of water [81].

It should be noted that, in the scaling method in Equations (12) and (13), using a reduced density $\left(\tilde{\rho }\right)$ of 3, the system freezes when $N_{m}\geq 10$. Fuchslin et al. [82] introduced a DPD scaling method that avoids freezing the system for single-component systems. Therefore, because our system is a binary mixture and falls on a scale where there is no freezing of the system, we employed the scaling method shown in Equations (12) and (13).

This parameterization scheme enabled the estimation of parameters from various experimental values, such as the number density of the components, the isothermal compressibility of the solvent, and the Flory–Huggins interaction parameter. For more detailed information on the parameterization scheme, please refer to Supplementary Section S4.3. The temperature-dependent water densities and isothermal compressibilities used to determine the DPD force parameters are listed in Supplementary Table S4. The resulting conservative force parameters in reduced units are provided in Supplementary Table S5.

### 3.3. The Nanogel Model

**Figure 8:**
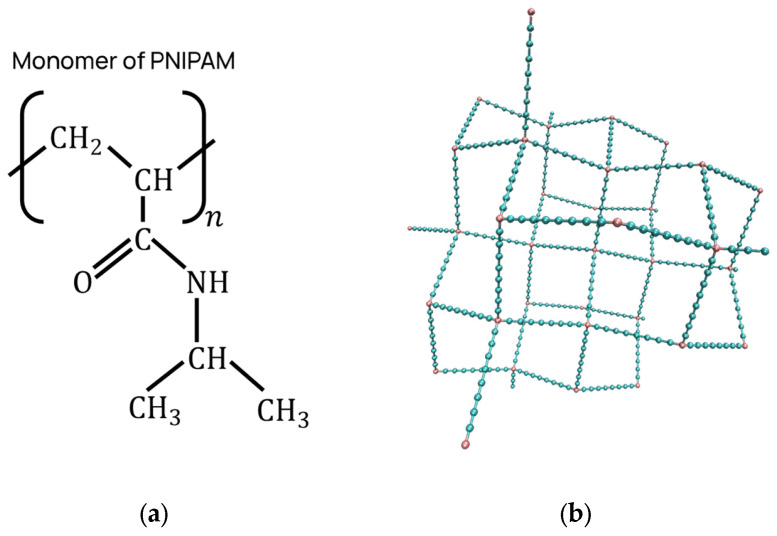
(**a**) Chemical structure of the N-isopropylacrylamide (PNIPAM) monomer. (**b**) Coarse-grained representation of the simulated neutral PNIPAM nanogel network. The nanogel is characterized by a diamond-like topology. In this structure, the inner crosslinkers (pink beads) are coordinated with four polymeric chains, while each inner chain is composed of eight monomer beads (cyan beads). The outermost crosslinkers are coordinated with one, two, or three chains, and the outermost chains contain up to eight monomers. The complete nanogel model consists of a total of 439 monomer beads.

The nanogel used in all simulations was a PNIPAM nanogel without any ionized monomers. It has a diamond-like topology, wherein all the inner crosslinkers are coordinated with four chains, and each inner chain comprises eight monomers. The outermost crosslinkers are coordinated with one, two, or three chains, and the outermost chains contain up to eight monomers. Figure 8 depicts the simulated PNIPAM nanogel along with the chemical formula of the PNIPAM monomers. Supplementary Section S1 contains detailed information on the simulated nanogel and its construction.

For simplicity, the crosslinkers had the same potential interactions as the nanogel monomers, differing only in the number of bonded interactions. The bonded interactions ($U_{bond}\left(r_{ij}\right)$) between adjacent particles were modeled using the following harmonic potential:

(14) $$U_{bond}\left(r_{ij}\right)=\frac{K}{2}{\left(r_{ij}-r_{0}\right)}^{2}$$

where K represents the elastic constant, and $r_{0}$ denotes the equilibrium bond length. In this model, K is set to 0.4 N/m and $r_{0}$ is set to 6.5 Å. The parameter values for the bound interactions were chosen to be consistent with those used by Quesada-Pérez et al. [32,33,35], enabling Langevin dynamics simulations to align closely with their results [33].

In this study, the water beads consisted of an aggregation of five water molecules ($N_{m}=5$) [83]. This level of coarse graining ensured that the volumes of both bead types were as similar as possible.

The cutoff distance of the model was obtained by imposing the value of the reduced density of the system $\tilde{\rho }$ (See Table 2), which was equal to 3 for all simulations [48].

(15) $$\tilde{\rho }=\rho_{w}\frac{r_{c}^{3}}{N_{m}m_{w}}$$

(16) $$r_{c}=\sqrt[{3}]{\frac{\tilde{\rho }N_{m}m_{w}}{\rho_{w}}}$$

where $m_{w}$ is the mass of a water molecule and $\rho_{w}$ is the experimental density of water at the simulated temperature. Consequently, the cutoff radius varies with temperature and serves as the length conversion factor to adjust the reduced length units for different simulated temperatures.

**Figure 9:**
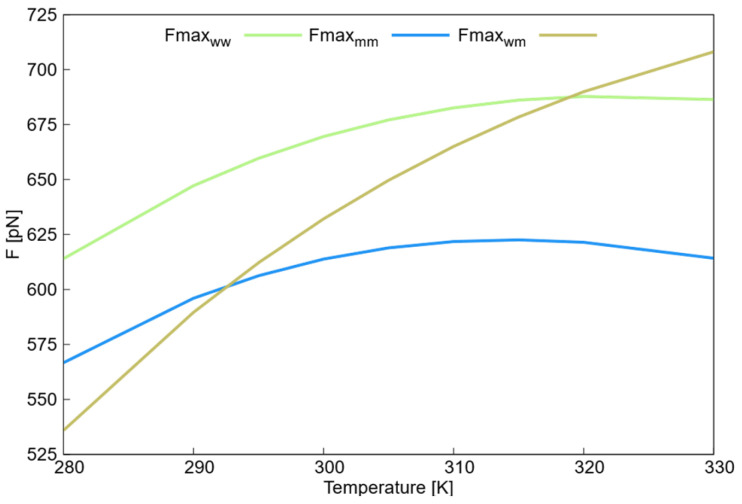
Temperature dependence of the DPD maximum repulsion parameters (Fmax*_ij_*) for the different conservative interactions. The plot illustrates the evolution of the force parameters for water–water (Fmax*_ww_*), monomer–monomer (Fmax*_mm_*), and water–monomer (Fmax*_wm_*) interactions across the simulated range of 280–330 K.

The maximum repulsion parameters of different DPD potentials as a function of temperature are plotted in Figure 9. The maximum repulsion parameters ${Fmax}_{mm}$, ${Fmax}_{ww}$, and ${Fmax}_{wm}$ increased with the temperature. However, although ${Fmax}_{mm}$ and ${Fmax}_{ww}$ remained constant after 305 K, ${Fmax}_{wm}$ continued to increase. This increase suggests that the collapse modeled through this force field was favored as the repulsion between the water and monomer beads increased. This specific balance of forces explains the induced hydrophobicity of the system: the monomers do not change chemically, but their ‘preference’ for self-interaction over solvation becomes the dominant structural driver. This behavior explains how the DPD simulation reproduced the collapse of the nanogel. At lower temperatures, the monomers interacted more stably with water molecules than with each other. As the temperature rose, the monomers became more stable and interacted with themselves rather than with water.

For more information on the temperature-dependent potential employed in Langevin dynamics simulations to mimic the hydrophobic effect, see Supplementary Section S2.1.

### 3.4. Reduced Units

The ESPResSo software lacked predefined unit systems to represent various magnitudes of the system. Consequently, a system of reduced units should be established. To achieve this, specific quantities of mass, length, and energy were selected to form the basis of the reduced unit system, from which other quantities, such as time, could be derived.

The reduced-unit systems used in the DPD simulations are presented in Table 2. This reduction in units involved adjusting the unit of mass by the mass of a water bead, the unit of distance by the cutoff distance of the interactions between beads, and the unit of energy by the energy of $k_{B}T$.

### 3.5. Computational Details

Nine DPD simulations in the range of 280–330 K were performed to reproduce the thermosensitive behavior of PNIPAM, along with nine Langevin dynamics simulations in the same temperature range. These simulations were conducted using ESPResSo software [73,74]. The radius of gyration was computed at every integration step using the ESPResSo function, and the radial distribution functions in the DPD simulations were computed using the ESPResSo function.

All simulations were performed in the NVT ensemble in a box with periodic boundary conditions, using the velocity Verlet integration algorithm. The simulation box for all DPD simulations was a cube with a side length of 22.5 $r_{c}$, containing 439 monomer beads and 33,620 water beads. The time step selected for the simulations was $∆t=0.01\tilde{t}$.

To achieve a faster thermalization of the system, a prior thermalization of $1\times 10^{6}$ time steps using Langevin dynamics was conducted. This was followed by DPD thermalization, consisting of $1\times 10^{6}$ time steps, and a production run consisting of $5\times 10^{6}$ time steps. For detailed information on the DPD simulations and their thermalization, see Supplementary Sections S3 and S4.

Langevin dynamics simulations were conducted using purely repulsive Weeks–Chandler–Andersen [84] potentials in conjunction with a temperature-dependent hydrophobic attractive potential between monomer beads, as described by Quesada-Pérez [33]. Detailed information on the Langevin simulations is provided in the Supplementary Section S2. The self-diffusion constants of water and the corresponding damping constants used in the Langevin dynamics simulations are reported in Supplementary Table S1. The reduced unit conversion and reduced damping constants are summarized in Supplementary Tables S2 and S3.

Similar to the DPD simulation, the simulation box for all Langevin dynamics simulations was a cube with a side length of 100$r_{c}$ containing 439 monomer beads. The time step chosen for the simulations was $∆t=0.01\tilde{t}$.

The Langevin dynamics simulations consisted of a thermalization process of $2.5\times 10^{6}$ time steps, followed by a production run of $5\times 10^{6}$ time steps.

The scripts needed to perform all simulations can be found at the GitHub repository: https://github.com/smadurga/DPD_nanogel, accessed on 1 January 2026.

## 4. Conclusions

The implementation of a dissipative particle dynamics (DPD) model with varying bead volumes, based on the Kacar et al. parameterization [60], allows for a more accurate consideration of steric effects, which are critical for capturing the high-density packing of the nanogel in its collapsed state. The parameterized DPD model with varying volume beads effectively investigated the swelling behavior of thermosensitive neutral nanogels, and the results were consistent with those of a previously established Langevin dynamics model [43]. In the DPD simulations, temperature-dependent hydrophobic interactions naturally emerge with the explicit inclusion of the solvent. Conversely, in Langevin dynamics, an attractive temperature-dependent potential must be incorporated between monomer beads to represent hydrophobic interactions, as water is implicitly treated. DPD simulations offer the advantage of explicitly simulating the effect of water, and because of their coarse-grained nature, they enable longer time-scale simulations than all-atom molecular dynamics, which is achieved by utilizing soft potential interactions between the beads.

This study demonstrated the volume phase transition of a neutral PNIPAM nanogel from an expanded state to a collapsed state with increasing temperature, as evidenced by the radii of gyration obtained from both DPD and Langevin dynamics simulations. The volume phase transition of PNIPAM with increasing temperature was also apparent from the analysis of various radial distribution functions obtained from DPD simulations. Moreover, the critical temperature of the volume phase transition was estimated to be 305–310 K, which is consistent with experimental data [64]. It should be noted that because of the nature of the bead potentials and the fact that the model parameters do not match directly, the values of the radius of gyration at different temperatures in the DPD simulations were lower than those obtained from Langevin dynamics.

The neutral PNIPAM nanogel model studied in this work did not include charged beads or ions from the ionic salts. For charged systems in DPD simulations, Slater-type charges [65] or Gaussian charge densities [66] must be considered.

## Figures and Tables

**Table 1:** Obtained mean radius of gyration with the associated standard error for both the DPD and Langevin dynamics simulations.

Temperature (K)	Langevin Dynamics Mean Radius of Gyration (Å)	DPD Mean Radius of Gyration (Å)
280	48.87 ± 0.23	44.00 ± 0.34
290	47.79 ± 0.21	42.45 ± 0.38
295	46.20 ± 0.23	39.89 ± 0.27
300	43.30 ± 0.19	37.59 ± 0.36
305	37.75 ± 0.20	33.15 ± 0.22
310	31.43 ± 0.14	26.13 ± 0.14
315	28.41 ± 0.04	22.37 ± 0.07
320	27.38 ± 0.04	20.88 ± 0.04
330	26.78 ± 0.03	19.98 ± 0.02

**Table 2:** Conversion systems of the reduced (dimensionless) units in the DPD simulations.

Quantities	Equation to Reduce the Quantities	Definition of Parameters
Mass	${\tilde{m}}_{i}=\frac{m_{i}}{m_{wbead}}$	$m_{wbead}=N_{m}m_{w}$
Length	$\tilde{r}=\frac{r}{r_{c}}$	$r_{c}=\sqrt[{3}]{\frac{\tilde{\rho }N_{m}m_{w}}{\rho_{w}}}$
Energy	$\tilde{U}=\frac{U}{k_{B}T}$	
Density	$\tilde{\rho }=\rho \frac{r_{c}^{3}}{m_{wbead}}$	
Time	$\tilde{t}=\frac{t}{r_{c}\sqrt{\frac{m_{wbead}}{k_{B}T}}}$	
Friction coefficient	$\tilde{\gamma }=\gamma \frac{r_{c}}{\sqrt{m_{wbead}k_{B}T}}$	

## Data Availability

The scripts needed to perform the simulations can be found at the https://github.com/smadurga/DPD_nanogel (accessed on 1 January 2026) GitHub repository.

## Supplementary Materials

The following supporting information can be downloaded at: https://www.mdpi.com/article/10.3390/ijms27031241/s1.
