# Supplementary material for: Temperature Dependence of a Thermosensitive Nanogel: A Dissipative Particle Dynamics Simulation of PNIPAM in Water
Source: Int J Mol Sci. 2026 Jan 26;27(3):1241. doi: 10.3390/ijms27031241 (PMC12897716; doi:10.3390/ijms27031241)
Supplement: Supplementary file 1 [file ijms-27-01241-s001.zip › ijms-4056524-supplementary.pdf]

Supporting Information

Temperature Dependence of a Thermosensitive Nanogel: A Dissipative Particle Dynamics Simulation of PNIPAM in Water

Daniel Valero, Francesc Mas * and Sergio Madurga *

Department of Material Science and Physical Chemistry, Institute of Theoretical and Computational Chemistry (IQTC), University of Barcelona (UB), 08028 Barcelona, Spain

* Correspondence: fmas@ub.edu (F.M.); s.madurga@ub.edu (S.M.)

S1. Nanogel generation

The generation of the nanogel involves three steps. The first step is to create the crosslinkers' coordinates. For this, the numpy and pyiron libraries for Python are used. The second step is to link the crosslinkers with chains composed of 8 monomer beads. This step is performed in the ESPResSo script that launches the Langevin dynamics simulations. Finally, a radius for the nanogel is chosen, and any bead outside that radius is deleted. This is also done in the ESPResSo script that launches the Langevin dynamics simulations.

S1.1 Nanogel crosslinkers generation

The script that generates the crosslinkers' coordinates is named "nanogel.py" [1]. This script creates the coordinates of the crosslinkers at the positions where carbon atoms would be in a diamond superlattice. These coordinates, once generated, are saved in a file named "positions.npy" [1].

S1.2 Nanogel chains generation

In the ESPResSo script used to launch the Langevin dynamics simulations (Langevin.py), there is a function called "build_chain" that creates the monomer chains linking the crosslinkers, adding the monomers' positions and the bonds.

S1.3 Nanogel size

Again, in the ESPResSo script used to launch the Langevin dynamics simulations (Langevin.py), there is a parameter called "cut_generation_dist"; this is the distance in reduced units from the center of the nanogel beyond which no monomer or crosslinker is placed. This parameter is used in the "Langevin.py" script and the "build_chain" function not to generate any crosslinkers or monomers, respectively, at positions outside of the "cut_generation_dist".

S2. Langevin dynamics simulations

The Langevin simulations consist of 439 monomer beads that interact with each other through two potentials: one repulsive and one attractive. The repulsive potential is a Weeks–Chandler–Andersen (WCA) potential, while the attractive potential employed is a temperature-dependent hydrophobic attractive potential between monomer beads, as described by Quesada-Pérez [2].

The ESPResSo script used to launch the Langevin dynamics simulations is called "Langevin.py" [1]. For it to work, the file "positions.npy" containing the coordinates of the crosslinkers must be in the same folder as the "Langevin.py" script. At the end of the simulation, a "Results.dat" file will be generated. This file contains the results of the simulations in four different columns: time step, radius of gyration (Å), total energy (kJ/mol), and temperature (K).

S2.1 Langevin dynamics Simulation Parameters

S2.1.1 Potential parameters

Eq. (S1) shows the WCA potential and its parameters. In this equation, r_{ij} is the distance between monomer beads, ε_{WCA} is the depth of the potential, σ is the effective monomer radius and r_c^{WCA} is the cut-off distance of the U_{WCA}.

$$U_{WCA}(r_{ij}) = \begin{cases} 4\varepsilon_{WCA}\left(\frac{\sigma^{12}}{r_{ij}{}^{12}} - \frac{\sigma^6}{r_{ij}{}^6} - c\right) & r_{ij} \le r_c^{WCA} \\ 0 & r_{ij} > r_c^{WCA} \end{cases} \quad \text{(S1)}$$

$$\sigma = 6.5\ \text{Å} \qquad r_c^{WCA} = \sigma 2^{1/6} \qquad \varepsilon_{WCA} = 2.475042\ KJ/mol$$

Eq. (S2) shows the hydrophobic attractive potential and its parameters. In this equation, r_{ij} is the distance between monomer beads, r_c^H is the cut-off distance of the $U_{hydrophobic}$ potential, the k_h is associated with the slope of the sigmoid, r_h is related to the range of the potential, and $E_h(T)$ is the temperature dependent function of the depth of the hydrophobic potential.

$$U_{hydrophobic}(r_{ij}, T) = \begin{cases} -\frac{E_h(T)}{2}\left(1 - tanh\left(k_h(r_{ij} - r_h)\right)\right) & r_{ij} \le r_c^H \\ 0 & r_{ij} > r_c^H \end{cases} \quad \text{(S2)}$$

$$r_h = 9\ \text{Å} \qquad k_h = \frac{10.99}{r_h}\text{Å}^{-1} \qquad r_c^H = 14.95\ \text{Å}$$

Eq. (S3) shows the temperature dependent function of the depth of the hydrophobic potential. In this equation, ε_{max} is the maximum depth of the $U_{hydrophobic}$ potential, $T_{\varepsilon/2}$ is the temperature at which $E_h(T) = \varepsilon_{max}/2$ and $k_{\varepsilon/2}$ is proportional to the slope of the function $E_h(T)$ at the point $T = T_{\varepsilon/2}$.

$$E_h(T) = \frac{\varepsilon_{max}}{2}\left(1 + tanh\left(k_{\varepsilon/2}(T - T_{\varepsilon/2})\right)\right) \quad \text{(S3)}$$

$$T_{\varepsilon/2} = 307.5\ K \qquad k_{\varepsilon/2} = 0.0667\ K^{-1} \qquad \varepsilon_{max} = 3.3121\ KJ/mol$$

Eq. (S2 and S3) are the equations that describes the added temperature-dependent potential employed to apply the hydrophobic effect that drives the nanogel to collapse.

S2.1.2 Langevin dynamics damping constant.

Eq. (S4) shows how the damping constant used in the Langevin dynamics simulations is calculated. In this equation, D_{water} is the self-diffusion constant of water and $M_{monomer}$ is the mass of the monomer beads. The self-diffusion constant of water is used as an approximation of the monomer bead diffusion in water. Since the aim of the work is not to obtain transport properties of the nanogel, the use of this approach does not imply a loss of accuracy. The self-diffusion constant of water and the calculated damping constants are shown in Table S1.

$$\gamma^{Lan}(T) = \frac{K_b T}{D_{water}\ M_{monomer}} \quad \text{(S4)}$$

$$M_{monomer} \approx 1.879066 \cdot 10^{-25}\ Kg$$

Table S1: Self-diffusion water constants at the different temperatures simulated and the calculated $\boldsymbol{\gamma^{Lan}}$.

T (K)	$\boldsymbol{D_{water}}$ $\boldsymbol{(10^{-9} m^2/s)}$	$\boldsymbol{\gamma^{Lan}(10^{12}/s)}$
280	1.3955	14.7355
290	1.8578	11.4640
295	2.1197	10.2208
300	2.4021	9.1721
305	2.7048	8.2814
310	3.0277	7.5194
315	3.3703	6.8640
320	3.7321	6.2970
330	4.5114	5.3720

S2.2 Langevin dynamics Reduced units

The conversion from real to reduced units for the Langevin dynamics simulations is shown in Table S2. Table S3 shows the reduced damping constants used in the simulations.

Table S2: Conversion system of the reduced units in the Langevin dynamics simulations.

Quantities	Equation to reduce the quantities
Mass	$\tilde{m} = \dfrac{m}{M_{monomer}}$
Length	$\tilde{r} = \dfrac{r}{\sigma}$
Energy	$\tilde{E} = \dfrac{E}{\varepsilon_{WCA}}$
Time	$\tilde{t} = \dfrac{t}{\sigma\sqrt{\dfrac{m_{mbead}\, N_A}{\varepsilon_{WCA}\, 10^3}}} = \dfrac{t}{\tau}$
Damping Constant	$\tilde{\gamma}^{Lan}(T) = \tau\gamma^{Lan}(T)$

Table S3: Reduced damping constants for each simulated temperature.

T (K)	$\boldsymbol{\tilde{\gamma}^{Lan}}$
280	64,7945
290	50,4091
295	44,9425
300	40,3311
305	36,4145
310	33,0643
315	30,1823
320	27,6890
330	23,6218

S3. Pre dissipative particle dynamics simulation

To achieve a faster thermalization of the system compared to thermalizing only with a DPD simulation, a prior thermalization simulation using Langevin dynamics is performed. This simulation uses the same parameters as the regular Langevin dynamics simulations. These simulations consist of 1×10^6 integration time steps at a temperature of 280 K. Once the thermalization is completed, the positions of the crosslinkers and monomers are saved in the file "id_type_pos.dat" [1] file, and the information of the bonds is stored in the file "id_bond_ids.dat" [1]. This thermalization is performed with the "DPD_Lan_Therm.py" [1] script.

The "id_type_pos.dat" and "id_bond_ids.dat" are then used in the DPD simulation script as starting point for the DPD simulations.

S4. Dissipative particle dynamics simulations

The ESPResSo script used to launch the DPD simulations is called "DPD.py" [1]. For it to work, the files "id_type_pos.dat" and "id_bond_ids.dat", containing the coordinates of the crosslinkers and monomer beads must be in the same folder as the "DPD.py" script. These DPD simulations were performed using a single core because, when using multiple cores, bond breaking appears.

At the end of the simulation a "Results.dat" file will be generated alongside with four other files containing the different radial function distributions measured. The "Results.dat" file contains the results of the simulations in four different columns, corresponding to time step, radius of gyration (Å), total energy (kJ/mol), and temperature (K). The files containing the results of the different measured radial distribution functions are as follows: "GDR_wm_Results.dat" containing the water-monomer radial distribution function, "GDR_ww_Results.dat" containing the water-water radial function distribution, "GDR_mm_Results.dat" containing the monomer-monomer radial function distribution and the "GDR_NMC_ww_Results.dat" containing the radial function distribution between water beads and the nanogel mass centre. All the results files of the radial function distributions have two columns, the first one contains the distance in angstroms and the second contains the value of the radial function distribution.

S4.1 DPD Simulation Parameters

S4.1.1 Physical parameters needed for the DPD simulations.

Table S4 contains the densities and isothermal compressibility of water used in the DPD simulations at different temperatures to obtain the different force parameters of the conservative interactions. The isothermal compressibility of water employed is from experimental data [3].

Table S4: Water density and isothermal compressibility of water used in the DPD simulations.

T (K)	$\rho_w\ (Kg/m^3)$	$\tilde{k}^{-1}\ (10^{-10}/Pa)$
280	999.87	4.9175
290	998.77	4.6739
295	997.80	4.5895
300	996.57	4.5248
305	995.10	4.4771
310	993.42	4.4441
315	991.53	4.4241
320	989.45	4.4158
330	984.79	4.4298

S4.2 Force parameters of the different conservative potential interactions in reduced units

Table S5 shows the force parameters in reduced units of the different conservative potential interactions. In this table, a_{ww} is the force parameter of the water-water conservative interaction, the a_{mm} is the force parameter of the monomer-monomer conservative interaction and the a_{wm} is the force parameter of the water-monomer conservative interaction.

Table S5: Force parameters in reduced units.

T (K)	a_{ww}	a_{mm}	a_{wm}
280	121.6040	112.2318	106.1304
290	123.8061	114.0117	112.7880
295	124.0837	114.0427	115.1539
300	123.9017	113.5908	116.9751
305	123.3142	112.7136	118.3124
310	122.3739	111.4704	119.2262
315	121.1278	109.9085	119.7677
320	119.6139	108.0715	119.9817
330	115.9322	103.7413	119.5995

S4.3 DPD Parametrization

1. Set the coarse-graining level of water beads such that $N_m < 10$. In our system $N_m = 5$ water molecules per bead.
2. Set the reduced density $\tilde{\rho} \geq 3$ and compute the cutoff distance: $r_c = \sqrt[3]{\frac{\tilde{\rho} N_m m_w}{\rho_w}}$. In our system $\tilde{\rho}$ is chosen equal to 3.
3. Compute the water-water force parameter a_{ww} using Groot and Warren parametrization [4]: $a_{ww} = N_m \frac{(\tilde{k}^{-1}-1)}{2\alpha\rho} \frac{k_B T}{r_c^4}, \alpha = 0.101$.
4. Substitute the obtained a_{ww} into the Kacar *et al.* [5] parametrization for beads of the same type: $a_{ww} = \frac{p - \rho_{w,pure} k_B T}{\alpha \rho_{w,pure}^2 r_c^4}, \alpha = 0.101$, then isolate the pressure p and compute its value $p = a_{ww} \alpha \rho_{w,pure}^2 r_c^4 + \rho_{w,pure} k_B T$.
5. Compute the monomer-monomer force parameter a_{mm} using Kacar *et al.* [5] parametrization for beads of the same type: $a_{mm} = \frac{p - \rho_{m,pure} k_B T}{\alpha \rho_{m,pure}^2 r_c^4}, \alpha = 0.101$
6. Compute the Flory-Huggins interaction parameter: $\chi_{ij}\,(T) = 0.5 + A\left(1 - \left(\frac{\Theta}{T}\right)\right), A = 35.2\ \Theta = 308.3$
7. Finally compute the water-monomer force parameter a_{wm} using Kacar *et al.* [5] parametrization for beads of different type: $a_{wm} = \sqrt{a_{ww} a_{mm}} + \frac{p}{0.0454(a_{ww}\rho_{w,pure} + a_{mm}\rho_{m,pure})} \frac{k_B T}{r_c^2} \chi_{wm}$.

S4.4 DPD Results

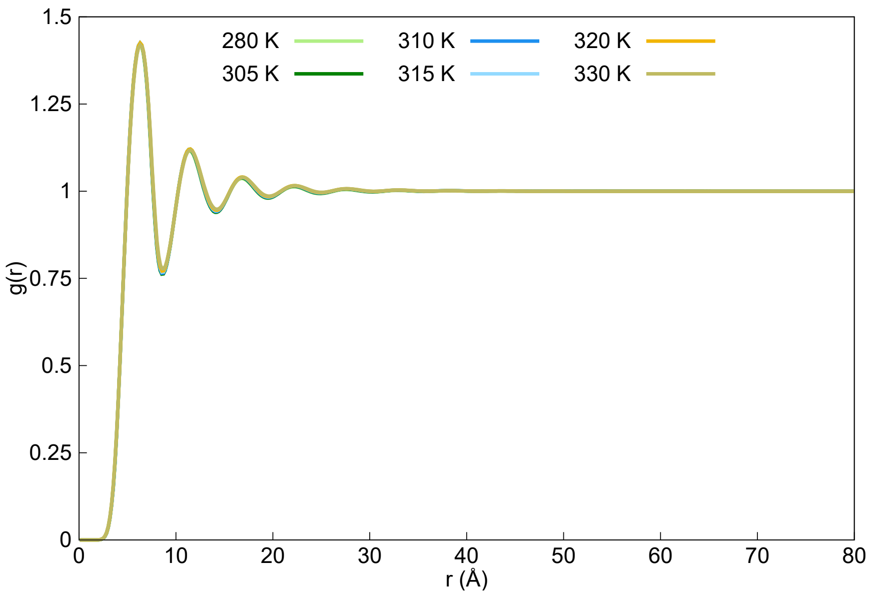

Figure S1. Radial distribution functions of DPD simulations of the water beads with themselves in the range of 280 to 330 K.

S5. References

[1] https://github.com/smadurga/DPD_nanogel GitHub repository

[2] M. Quesada-Pérez, J. Ramos, J. Forcada, A. Martín-Molina, Computer simulations of thermo-sensitive microgels: Quantitative comparison with experimental swelling data, Journal of Chemical Physics 136 (2012). https://doi.org/10.1063/1.4729946.

[3] R.A. Fine, F.J. Millero, Compressibility of water as a function of temperature and pressure, J Chem Phys 59 (1973) 5529–5536. https://doi.org/10.1063/1.1679903.

[4] R.D. Groot, P.B. Warren, Dissipative particle dynamics: Bridging the gap between atomistic and mesoscopic simulation, Journal of Chemical Physics 107 (1997). https://doi.org/10.1063/1.474784.

[5] G. Kacar, E.A.J.F. Peters, G. De With, A generalized method for parameterization of dissipative particle dynamics for variable bead volumes, EPL 102 (2013). https://doi.org/10.1209/0295-5075/102/40009.
